# Supplementary material for: Non-universal transmission phase behaviour of a large quantum dot
Source: Nat Commun. 2017 Nov 22;8:1710. doi: 10.1038/s41467-017-01685-z (PMC5700201; doi:10.1038/s41467-017-01685-z)
Supplement: Supplementary file 1 — Supplementary Information [file 41467_2017_1685_MOESM1_ESM.pdf]

Supplementary Figures

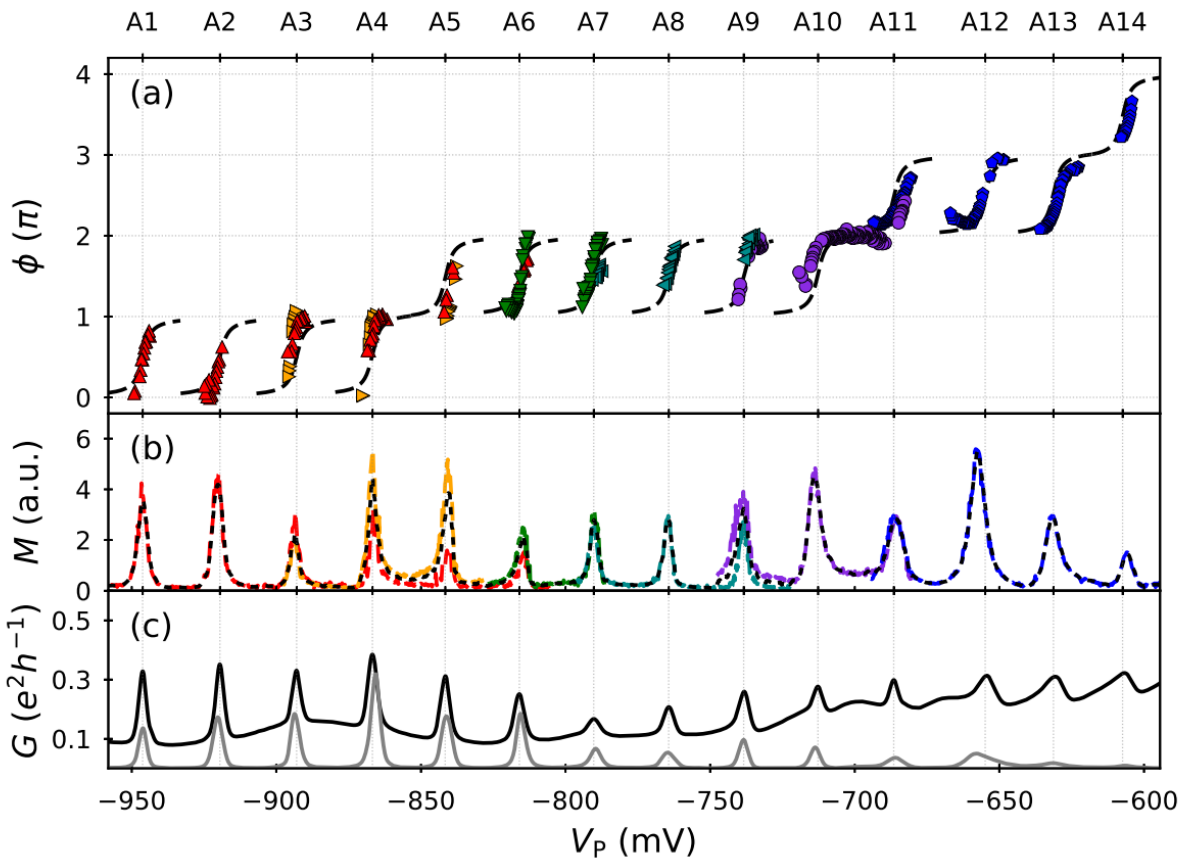

Supplementary Figure 1. **Transmission phase data sets along fourteen resonances.** (a) Transmission phase, Φ, constructed from six measurements. The individual data sets are indicated via different symbols and colors. The dashed black line in the background is constructed from measurements of conductance, G, along the resonances and is used for data alignment and as guide to the eye in the main paper. (b) Magnitude of AB oscillations, M, obtained from Fourier analysis of the individual measurements (colored lines). The black, densely dashed line is constructed by convolution of the data sets by a Hann function and is used to represent an approximate course. (c) Electrical conductance, G, measured along the investigated resonances at the lower terminal of the interferometer (black line). The conductance in between the resonances stems from transmission through the upper interferometer branch. In addition the bare conductance through the quantum dot is shown (grey line), where the electrons are steered only through the lower interferometer branch.

Supplementary Notes

Supplementary Note 1: Construction of guide to the eye for transmission phase data

In Breit-Wigner resonance theory the transmission phase experiences an arctangent shift: Φ = arctan((V_P–$V_{P,0}$)/ΔV_{HWHM}). Here $V_{P,0}$ is the central position of the resonance and ΔV_{HWHM} is the half width at half maximum of the peak. By fitting the Coulomb blockade peaks with Lorentzian functions one can obtain $V_{P,0}$ and ΔV_{HWHM} and construct an approximate transmission phase course. This approach is not profound but provides a reasonable guide to the eye that is presented in the main paper. The expected course is constructed on the basis of conductance data measured at the lower terminal of the interferometer – see black line in Supplementary Figure 1c. Fitting each peak with a Lorentzian function, we obtain the positions of the maxima, $V_{P,0}$, and an averaged value of ΔV_{HWHM}. Using those fit parameters then we construct an approximately expected phase shift for each resonance – see dashed line in Supplementary Figure 1a. In between the resonances phase lapses of π are introduced according to the transmission phase measurements.

By closing the tunnel barriers, the electrons are steered only through the lower interferometer branch in the Coulomb diamond measurements. As the tunnel barriers are closed – a voltage change of approximately 300 mV – the QD states can be affected due to capacitive crosstalk. Therefore, the Coulomb blockade peaks in this condition are not appropriate for the construction of the guide to the eye and for the comparison with the AB oscillation magnitude. In Supplementary Figure 1c the bare conductance through the QD is shown (grey line). Comparing the bare conductance through the QD (grey line) with the conductance at normal interferometer operation (black line), small deviations of the CP positions are apparent, which stem from the aforementioned crosstalk. In Fig. 4 of the main manuscript, therefore, we use the conductance measured at the lower terminal in normal interferometer mode, where the electrons can pass through both branches.

Supplementary Note 2: Data alignment along fourteen successive resonances

Changing V_P over a large range affects the coupling of the quantum dot to the two leads in lower interferometer branch. This crosstalk reduces at some point the visibility of the AB oscillations. In order to scan also through the resonances of low AB oscillation visibility, we split the measurement and compensate the visibility loss by retuning the interferometer configuration. To align the fine-tuned data sets, it is necessary that the measurements have overlapping V_P intervals. The data alignment regarding plunger gate voltage, V_P, is performed by overlapping the AB oscillation peaks. The alignment regarding phase is performed manually using the guide to the eye (see Supplementary Note 1). Following this approach with six measurements we can construct the course of the transmission phase along a set of fourteen successive resonances. Supplementary Figure 1 shows transmission phase and AB oscillation magnitude data for each of the conducted measurements – indicated via different colors and symbols. To obtain an approximate course of the AB oscillation magnitude along all of the resonances that is presented in the main paper, we convolute the corresponding M data sets shown in Supplementary Figure 1b with a Hann function – see black, densely dashed line.
